# Improved conditional expression systems resulting in physiological level of HNF4α expression confirm HNF4α induced apoptosis in the pancreatic β-cell line INS-1

**DOI:** 10.1186/1756-0500-2-210

**Published:** 2009-10-17

**Authors:** Sabine Senkel, Christoph Waldner, Gerhart U Ryffel, Heike Thomas

**Affiliations:** 1 Institut für Zellbiologie (Tumorforschung), Universitätsklinikum Essen, Universität, Duisburg-Essen, D-45122 Essen, Germany

## Abstract

**Background:**

To analyze gene function in mammalian cells tetracycline inducible expression of a gene-of-interest at a specific genomic location (Flp-In T-REx™) is most attractive. However, leakiness of basal transgene expression and artificially high expression level upon tetracycline addition may be disadvantageous.

**Findings:**

To solve these problems, we developed two different approaches to improve our pancreatic β-cell line INS-1 Flp-In T-REx™ expressing the tissue restricted transcription factor HNF4α under control of tetracycline. On the one hand we replaced the strong full length CMV promoter (CMV-Wt) with a weaker 5'-deleted CMV promoter fragment of 138 nucleotides in length (CMV-138). On the other hand we extended our INS-1 Flp-In T-REx™ cell lines with a Shield-1 dependent conditional control system of protein stability. Therefore, we fused HNF4α to the destabilization domain (DD) deduced from human FKBP12 protein. As a result in both approaches basal transgene expression level was markedly reduced, but HNF4α induction could still be maintained. Additionally, we could show that a low increase in HNF4α induces caspase activity indicating an apoptotic effect of HNF4α in these cells.

**Conclusion:**

In the present study we considerably improved our INS-1 Flp-In T-REx™ cell lines conditionally expressing HNF4α to reduce leakiness and to optimize exogenous HNF4α protein expression to a physiological level. As an important result we could extend our previous results that HNF4α induces apoptosis in the pancreatic β-cell line INS-1 with the new aspect that an expression level of the HNF4α transgene marginally exceeding the endogenous level is sufficient to trigger apoptosis.

## Background

Stable integration of inducible transcription factors is widely used to analyze gene function in mammalian cells. Among others the most commonly used system is the tetracycline inducible expression system. Based on repression by the Tet-repressor (TetR) the Flp-In T-REx™ system (Invitrogen) uses a full-length CMV promoter that contains two *tetO *sequences in tandem and a genomic integrated FRT site that can be used to integrate any gene-of-interest by Flp recombinase mediated integration. A limitation of this system may consist in considerable background expression without the activator tetracycline and in artificially high level of protein expression upon activation (our data).

Recently, another inducible system was developed that allows conditional protein degradation [1]. The human FK506- and rapamycin-binding protein (FKBP12) is rapidly and constitutively degraded in mammalian cells. Protein fusion of this destabilizing domain (DD) transfers the instability and addition of the ligand Shield-1 that binds to the destabilizing domain, protects the fusion protein from rapid degradation [1].

The rat pancreatic β-cell line INS-1 has retained many properties of β-cells including glucose induced insulin secretion [2] and is frequently used for conditional expression of introduced genes by the Tet-inducible system (for references see [3]). However, to overcome clonal differences by random genomic insertion, we have established the Flp-In T-REx™ system (Invitrogen) [3] to conditionally express transcription factors in β-cells [3-5].

The nuclear receptor hepatocyte nuclear factor 4α (HNF4α) is expressed as isoforms by alternative splicing and differential promoter usage (P1 and P2 promoter) [6]. P2 derived transcripts are predominantly expressed in mammalian β-cells and corresponding cell lines [7-9]. Heterozygous mutations in the human *HNF4α *gene lead to maturity-onset diabetes of the young subtype 1 (MODY1) [6] and there is evidence that *HNF4α *is also a susceptibility gene for common type 2 diabetes [10]. Defective regulation by HNF4α has been assumed to contribute to impaired glucose stimulated insulin secretion in diabetic patients [6]. Additionally, we showed that inducible HNF4α in INS-1 cells changes cell morphology, decreases proliferation and increases apoptosis [5]. The splice variant HNF4α2 was more efficient than HNF4α8 [5] reflecting the additional activation domain present in the HNF4α2 protein derived from the P1 promoter [6].

In the present study we improved our INS-1 Flp-In T-REx™ cell lines to reduce basal transgene expression in the absence of tetracycline and to limit induced HNF4α expression to a physiological level.

## Results

### The Flp-In INS-1 cell lines conditionally expressing HNF4α are markedly leaky

**Figure 1:**
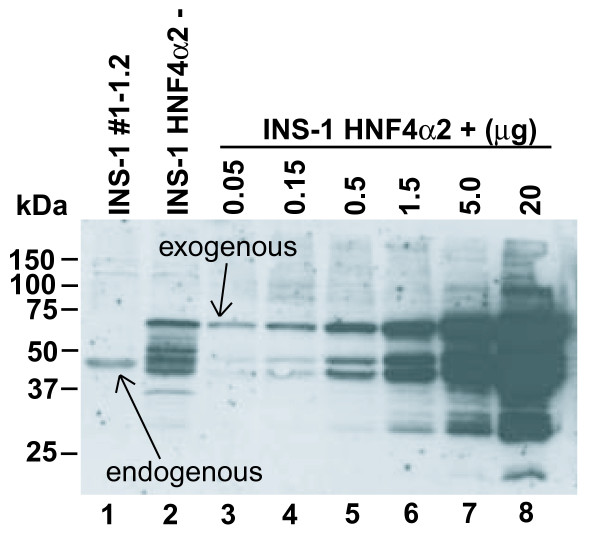
**Western blot analysis of INS-1 cell extracts**. Whole cell extracts of the indicated Flp-In INS-1 cell lines were analyzed by Western blotting using the HNF4α-(C-19)-antibody (Santa Cruz) recognizing P1 as well as P2 promoter derived proteins. The cell line INS-1 HNF4α2 [5] was cultured without (-) or with (+) 50 ng/ml tetracycline for 24 h. 20 μg of total protein were loaded. For the induced cell line (+), different amounts of whole cell proteins were used to estimate the fold induction of the transgene. The exogenous HNF4α protein was cloned C-terminally to six myc tag repeats. Therefore, the myc-HNF4α protein has an increased molecular mass of approximately 10 kDa in comparison to the endogenous protein. This results in a considerable shift in the western blot. The unexpected higher mobility of some minor bands of the exogenous HNF4α proteins possibly reflects degradation products.

Recently, we could improve the Western blot analysis to compare the expression levels of the endogenous and exogenous HNF4α protein in INS-1 cell extracts. As shown in Figure 1 (lane 1) our Flp-In INS-1 cell clone 1-1.2 showed endogenous expression of the HNF4α protein, which can be distinguished from the 78 amino acids larger exogenous protein carrying an N-terminal myc-tag (lane 2-8). In the uninduced state basal HNF4α transgene expression markedly exceeds the expression level of the endogenous HNF4α gene (compare lanes 1 and 2). Upon induction by tetracycline we estimate an additional 80-fold up-regulation but such a high overexpression could result in unphysiological effects of HNF4α in our Flp-In INS-1 cells.

### Flp-In INS-1 cell lines conditionally expressing HNF4α from a 5' deleted CMV promoter are less leaky

**Figure 2:**
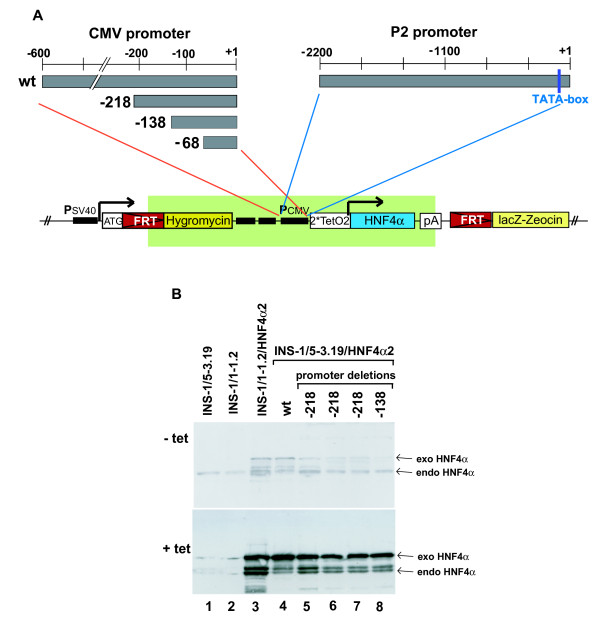
**Improved Flp-In INS-1 cell lines**. (**A**) Schematic presentation of the vector construct to generate Flp-In INS-1 cell lines conditionally expressing HNF4α under control of the 5'-deleted CMV promoter (left) or the P2 promoter (P2/-2200 from [7]) from the human HNF4α gene (right). Both promoters are tetracycline inducible due to the operator sites (2*TetO2) upstream of the transcription start site and the constitutively expressed tetracycline repressor (TetR) [3]. (**B**) Western blotting of whole cell extracts of the indicated Flp-In INS-1 cell lines with the HNF4α-antibody C-19 (Santa Cruz) to detect the HNF4α2 splice variant expressed from the full length or 5' deleted CMV promoter. Cells were cultured without (- tet) or with (+ tet) 50 ng/ml tetracycline for 24 h. 18 μg of total protein were loaded per lane and the signal for the exogenous (exo) as well as endogenous (endo) HNF4α is marked. Since the Flp-In INS-1 cell line #1-1.2 used previously [3] has lost its glucose induced insulin secretion (data not shown), we used now the cell line #5-3.19 that retains glucose sensitivity (data not shown) and that shows an identical expression level of endogenous HNF4α (lanes 1 and 2). The observation of differential characteristics in the INS-1 cells has been observed in our initial study [3] and reflects a property typical of the INS-1 cell line [23].

**Figure 3:**
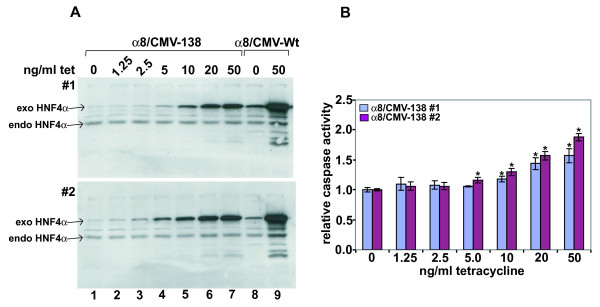
**Induction of apoptosis upon expression of HNF4α8**. (**A**) Western blotting of whole cell extracts of the INS-1 α8/CMV-138 cell lines (#1 and #2) and the α8/CMV-Wt cell line with the HNF4α-antibody C-19 (Santa Cruz). Cells were cultured with the indicated concentrations of tetracycline for 3 days. 20 μg of total protein were loaded per lane and the signal for the exogenous (exo) as well as endogenous (endo) HNF4α is marked. (**B**) Caspase 3/7 activity assay. The indicated INS-1 cell lines were treated with the indicated tetracycline concentrations for 3 days, lysed and caspase 3/7 activity was assessed. The relative caspase activity was calculated using the appropriate uninduced cells as a reference. Data are mean ± SD of six determinations and significant differences (p-value < 0.01) are indicated with an asterisk.

To decrease the basal HNF4α2 transgene expression in our Flp-In INS-1 cell lines we replaced the full length CMV promoter (CMV-Wt) with 5'-deleted CMV promoter fragments of 218 (CMV-218), 138 (CMV-138) or 68 (CMV-68) nucleotides in length (Figure 2A). Using the CMV-68 construct we failed to establish stable cell lines, possibly due to the loss of an enhancer activity acting on the hygromycin resistance gene as well. For cell lines with CMV-Wt, CMV-218 and CMV-138 constructs basal transgene expression was dependent on the CMV promoter length (Figure 2B, top, lane 3-8) with CMV-138 having the lowest activity. Induction with tetracycline resulted in an increased HNF4α transgene expression in each cell line (Figure 2B, bottom, lanes 3-8). Based on these data we used the CMV-138 promoter to establish cell lines expressing the HNF4α8 or HNF4α2 isoform derived from the P2 and P1 promoter, respectively. Figure 3A confirms the reduced basal transgene expression for the cell lines α8/CMV-138#1 and #2 in comparison to CMV-Wt (lanes 1 and 8). In both cell lines transgene induction is dependent on tetracycline concentration (Figure 3A, lane 2-7). Expression of the transgene is comparable to the expression of the endogenous HNF4α at 5 ng/ml tetracycline for cell line #1 (lane 4, top) and 2.5 ng/ml tetracycline for cell line #2 (lane 3, bottom).

Investigating the level of HNF4α protein inducing apoptotic events we observed a significant increase in caspase activity starting at a concentration of 5 to 10 ng/ml tetracycline (Figure 3B). At this concentration the expression level of the HNF4α8 transgene just starts to exceed the endogenous level of HNF4α (Figure 3A and 3B). The cell lines containing the HNF4α2 transgene have most similar properties (data not shown).

In conclusion, our improved experimental system shows that even a small increase in HNF4α is sufficient to induce apoptotic effects in the pancreatic β-cell line INS-1.

### Long-term induction of the HNF4α transgene leads to its downregulation

Upon long-term induction of the INS-1 cell line α2/CMV-138#1 with 50 ng/ml tetracycline we observed a marked decrease in HNF4α transgene expression. As shown by immunostaining (additional file 1, Figure S1), induction for 2 days resulted in transgene expression in 70% of the cells, whereas this number was dramatically diminished to 53%, 4% and 9% after 7, 14 and 23 days of induction, respectively. We observed this phenomenon also for the cell lines α2/CMV-138#2 and α2/CMV-Wt (data not shown) indicating a silencing of the CMV promoter that is independent of its length.

### Cell lines conditionally expressing a ligand-controlled HNF4α8 fusion protein driven by a tetracycline inducible P2 promoter activates caspases

**Figure 4:**
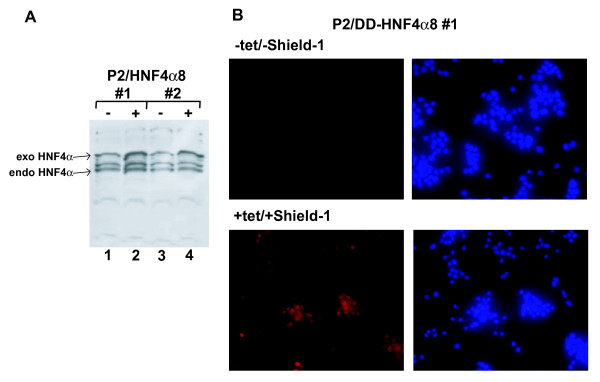
**Expression of HNF4α8 under the control of the P2 promoter**. (**A**) Western blotting of whole cell extracts of the INS-1 P2/HNF4α8 cell lines (#1 and #2) with the HNF4α-antibody C-19 (Santa Cruz). Cells were cultured without (-) or with (+) 50 ng/ml tetracycline for 24 h. 20 μg of total protein were loaded per lane and the signal for the exogenous (exo) as well as endogenous (endo) HNF4α is marked. (**B**) Immunofluorescence of INS-1 P2/DD-HNF4α8#1 cells without (upper panel) or with (lower panel) induction with 50 ng/ml tetracycline and 1 μM Shield-1 for 45 h. HNF4α was detected using a myc-tag specific primary antibody and a Cy3-coupled secondary antibody (red). The cells were also stained with DAPI (blue) to visualize the total number of cells. The fusion protein is located in the nucleus comparable to the wild type HNF4α protein.

**Figure 5:**
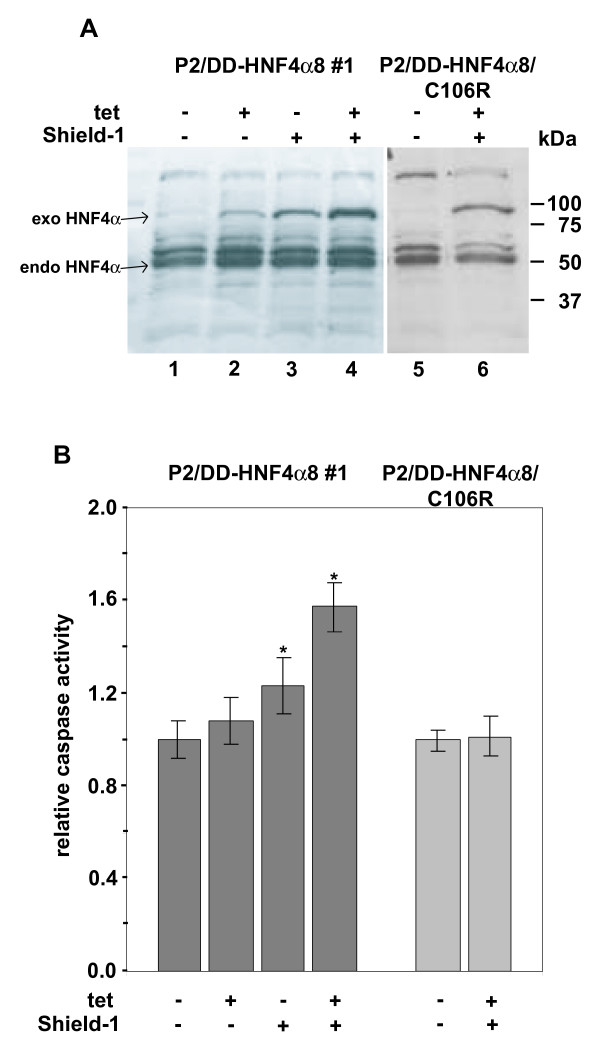
**Conditional expression of the DD-HNF4α fusion protein**. (**A**) Western blotting of whole cell extracts of the INS-1 P2/DD-HNF4α8 wild type #1 and C106R-mutant cell line with the HNF4α-antibody C-19 (Santa Cruz). Cells were cultured without (-) or with (+) 50 ng/ml tetracycline and/or 1 μM Shield-1 for 5 days. 15 μg of total protein were loaded per lane and the signal for the exogenous (exo) as well as endogenous (endo) HNF4α is marked. (**B**) Caspase 3/7 activity assay. The cells were treated with 50 ng/ml tetracycline and/or 1 μM Shield-1 for 5 days, lysed and caspase 3/7 activity was assessed. The relative caspase activity was calculated using the appropriate uninduced cells as a reference. Data are mean ± SD of six determinations and significant differences (p-value < 0.01) are indicated with an asterisk.

As the CMV promoter is downregulated upon long-term induction, we generated a Flp-In INS-1 cell line expressing HNF4α8 under control of a tetracycline inducible HNF4α P2 promoter carrying the tet-operator immediately downstream of the TATA box (Figure 2A). As shown in Figure 4A for two independent cell lines, basal HNF4α transgene expression is high without induction (lane 1 and 3) and only marginally increased upon addition of tetracycline (lane 2 and 4). To improve inducibility, we fused the L106P mutant of the human FKBP12 protein (the destabilizing domain, DD) [1] N-terminal to the myc-tagged HNF4α8 protein. As shown in Figure 4B, lower panel, generation of stable cell lines resulted in high expression of the DD-HNF4α8 fusion protein in more than 70% of the cells upon induction with tetracycline and Shield-1, but in the absence of both inducers there is no fusion protein detectable (Figure 4B, upper panel). This is confirmed by Western blot analysis (Figure 5A) showing that expression of the fusion protein is only weak after addition of tetracycline or Shield-1 alone (lanes 2 and 3), but markedly increased upon addition of both inducers simultaneously (lane 4).

To prove the functional properties of the DD-HNF4α protein we measured the executioner caspases 3 and 7, using DD-HNF-4α8 wild type in comparison to the C106R mutant protein, known to impair the DNA binding of HNF4α [5]. Figure 5B shows that induction of DD-HNF4α8 wild type with tetracycline and Shield-1 resulted in a significant increase in caspase activity that was absent in the DD-HNF4α8-C106R mutant. Obviously, the magnitude of induction of caspase activity correlates with the expression level of the DD-HNF4α8 wild type protein (compare panel A and B in Figure 5). In conclusion, our results demonstrate that the DD-HNF4α8 wild type protein is functional and that a small increase in HNF4α induces caspase activity in the pancreatic β-cell line INS-1.

## Discussion

To reduce high basal transgene expression in the absence of tetracycline (Figure 1, lane 2) and to allow induction at physiological levels, we decreased the strength of the CMV promoter by deleting enhancing elements [11] in the INS-1 Flp-In T-REx™ cell lines that conditionally express HNF4α [3,5]. For our experiments the CMV-138 promoter construct was optimal as the basal activity was reduced to a level below endogenous HNF4α expression, but still gave several fold induction (Figure 2 and 3). The most suitable CMV promoter deletion must be chosen for each experiment, as in HEK293 cells conditional expression of HNF4α showed a low background even using the full-length CMV promoter [12].

In the second approach we constructed a destabilized DD-HNF4α fusion protein that could successfully be stabilized by addition of Shield-1. This system seems to be applicable for many different proteins [1,13-16] and we used it, since tetracycline induced expression using the P2 promoter was inefficient (Figure 4A). We could prove that the DD-HNF4α fusion protein retains its biological property, because it induces apoptosis in INS-1 cells upon Shield-1 addition (Figure 5B) and transactivates a luciferase reporter gene driven by the human HNF1-promoter containing one HNF4 binding site (data not shown).

Upon long-term induction of the CMV promoter by tetracycline we observed silencing of transgene expression which did not occur, if the cells were cultured without tetracycline. It is likely that this inactivation does not encompass the entire integration site, as the cells are grown continuously in the presence of hygromycin. Progressive silencing of stable integrated transcription units containing the human CMV immediate-early promoter/enhancer has been reported previously [17]. Our data show that the P2 promoter of the HNF4α gene is not silenced upon long-term induction (data not shown) demonstrating that silencing of transgenes is dependent on the promoter type as previously reported [18,19].

To get a tetracycline-inducible P2 promoter we inserted the tet operator sequences just downstream of the TATA box in analogy to the CMV promoter. For unknown reasons this modified P2 promoter is poorly inducible by tetracycline. We are not aware of successful tetracycline regulation for any polymerase II promoter except the CMV promoter, although tetracycline control of RNA III polymerase promoters is well established [20].

Applying two improved conditional systems we extended our previous results by showing that even a small increase in HNF4α is sufficient to induce apoptosis in the pancreatic β-cell line INS-1 [5]. A functional role of HNF4α in apoptosis seems to be a β-cell restricted effect, as overexpression of HNF4α in hepatoma cells [21], embryonic F9 cells [22] as well as in HEK293 cells [12] exclusively affects cell proliferation. Whether this apoptotic effect of HNF4α plays an essential role in the endocrine pancreas in vivo, is presently unknown.

## Methods

### Plasmid constructs

Details are given in additional file 2.

### Establishment of INS-1 Flp-In T-REx cell lines

Establishment and culturing of the Flp-In T-REx INS-1 host cell lines used, #1-1.2 and #5-3.19, was as previously described [3]. Stable INS-1 Flp-In T-REx cell lines carrying the inducible transgenes were generated essentially as described in the Flp-In™ T-REx™ Core Kit Manual (Invitrogen). Co-transfection of the Flp expression vector pCSFLPe with the pcDNA5/FRT/TO vector containing the gene-of-interest was carried out using lipofectamine (Fa. Invitrogen) and hygromycin-B (Fa. Roth) (150 μg/ml) selection.

### Western blotting and immunofluorescence

The anti-myc tag antibody 9E10 was used for detection of myc-conjugated proteins, and the HNF4α-(C19)-antibody (Santa Cruz, Heidelberg, Germany, Nr. sc-6556) was employed for detection of HNF4α. For Western blots, peroxidase-coupled monoclonal mouse-anti-goat/sheep IgG, Clone GT-34 (Sigma-Aldrich, Saint-Louis, Missouri, USA, Nr. A9452) was employed as secondary antibody for the detection of HNF4α using the ECL system (Amersham Biosciences). For immunofluorescence, Cy3-conjugated rat anti-mouse [Dianova, Hamburg, Germany; F(ab')2-fragment, #415-166-166] was used as secondary antibody for detection of myc.

### Caspase activity

Caspase 3 and 7 activity was measured using the Caspase-Glo™ 3/7 Assay from Promega (Cat.No. G8090). Cells were plated at a density of 30.000 cells/well for the assay after 3 days or 10.000 cells/well for the assay after 5 days in white-walled 96-well plates. Prior to measurement in a luminometer (GENios multimode research reader; Tecan, Crailsheim, Germany) cells were incubated with Caspase-Glo™ 3/7 reagent for 1 h.

## Competing interests

The authors declare that they have no competing interests.

## Authors' contributions

SS carried out the entire experimental work. CW participated in the theoretical and experimental design of the protein stability experiments. GUR and HT conceived the study. GUR participated in the design of the study and contributed to draft the manuscript. HT coordinated the study, participated in the design of the experiments, analyses of data and was in charge of writing the final version of the manuscript. All authors read and approved the final manuscript.

## Supplementary Material

Additional file 1 **HNF4α2 expression upon long-term induction**. Immunofluorescence of Flp-In INS-1 α2/CMV-138#1 cells after induction with 50 ng/ml tetracycline. Click here for file

Additional file 2 **Plasmid constructs (Methods)**. The text provided describe the cloning of all plasmid constructs used in this study. Click here for file
