# Supplementary material for: Improved conditional expression systems resulting in physiological level of HNF4α expression confirm HNF4α induced apoptosis in the pancreatic β-cell line INS-1
Source: BMC Res Notes. 2009 Oct 17;2:210. doi: 10.1186/1756-0500-2-210 (PMC2768738; doi:10.1186/1756-0500-2-210)
Supplement: Additional file 1 — HNF4α2 expression upon long-term induction. Immunofluorescence of Flp-In INS-1 α2/CMV-138#1 cells after induction with 50 ng/ml tetracycline. [file 1756-0500-2-210-S1.PDF]

Additional file 1

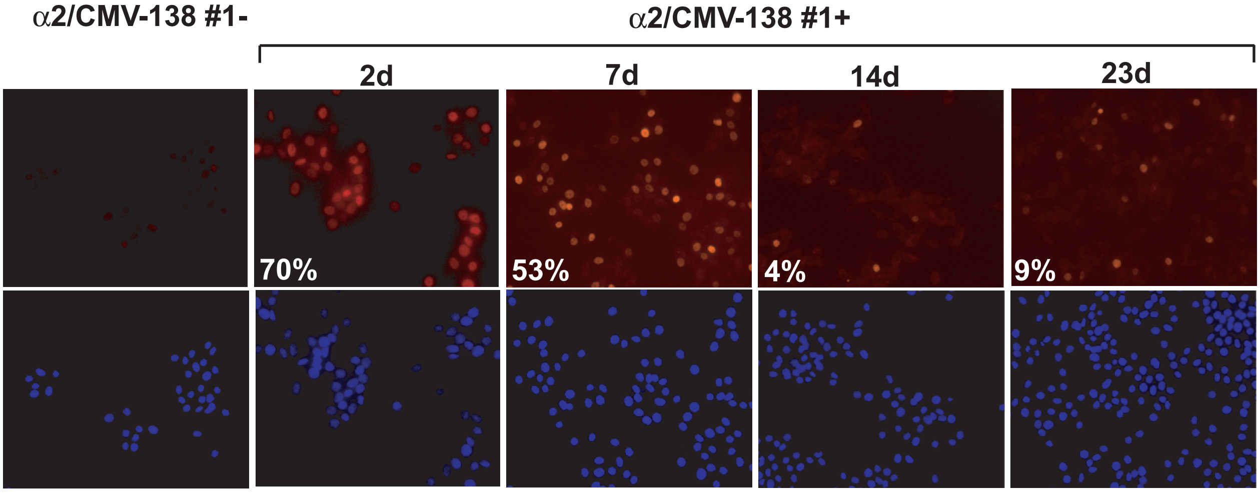

Figure S1 -HNF4α2 expression upon long-term induction

Immunofluorescence of Flp-In INS-1 α2/CMV-138#1 cells after induction with 50 ng/ml tetracycline for the indicated time periods (d: days). For these long-term experiments the cell lines were expanded by trypsinisation. HNF4α was detected using a myc-tag specific primary antibody and a Cy3-coupled secondary antibody (red). The cells were also stained with DAPI (blue) to visualize the total number of cells.
