# Supplementary material for: Improved conditional expression systems resulting in physiological level of HNF4α expression confirm HNF4α induced apoptosis in the pancreatic β-cell line INS-1
Source: BMC Res Notes. 2009 Oct 17;2:210. doi: 10.1186/1756-0500-2-210 (PMC2768738; doi:10.1186/1756-0500-2-210)
Supplement: Additional file 2 — Plasmid constructs (Methods). The text provided describe the cloning of all plasmid constructs used in this study. [file 1756-0500-2-210-S2.PDF]

Additional file 2

Plasmid constructs

5'-deleted CMV promoter constructs were generated by introducing PCR fragments into the *MluI* and *HindIII* restriction sites of the pcDNA5/FRT/TO vector (Invitrogen, Karlsruhe, Germany) carrying either the HNF4α2 or the HNF4α8 transgene [1]. PCR was performed with a set of forward primers corresponding to the 5'-end of the indicated promoter sequence and the reverse primer 5'-ATCCGAGCTCGGTACCAAGC-3', introducing *MluI* and *HindIII* restriction sites, respectively.

For the tet-inducible P2 promoter construct a promoterless intermediate of the pcDNA5/FRT/TO vector carrying the HNF4α8 transgene was constructed introducing a *MluI* restriction site upstream of the tet-operator (TetO2) sequence. Therefore, PCR was performed on the vector specified above with the forward primer 5'-ACGTTTACGCGTCTCCCTATCAGTGATAGAG-3' and the reverse primer 5'-TGGATCCGAGCTCGGTAC-3' and introduced as *MluI-HindIII* restriction fragment into pcDNA5/FRT/TO/HNF4α8 thus replacing the CMV promoter sequence. The final construct was generated by introducing a PCR fragment of the human P2 promoter region from -2073 to -23 upstream of the presumed transcription initiation site [2] into the *MluI* restriction site of the intermediate. PCR was performed with the forward primer 5'-ACGTTTACGCGTGGAAGGCAATGTGAGACC-3' and the reverse primer 5'-ACGTTTACGCGTTTATCTTATTGATTCTTCTAATC-3', introducing a *MluI* restriction site on both sites of the promoter sequence.

The genetic fusion construct (DD-HNF4α8) of the L106P mutant of the FKBP destabilization domain [3] and the myc-HNF4α8 was generated in three cloning steps: **1.** PCR of the HNF4α8 sequence was performed on the vector pcDNA5/FRT/TO/P2-HNF4α8 with the forward primer 5'-CGGAAGAATTCGTCAGCGTGAACGCGCC-3' and the reverse primer 5'-ACGGGCCCTCTAGACTCG-3', introducing an *EcoRI* restriction site. The *EcoRI-NotI* digested PCR fragment was used to replace YFP-HA in pBMN L106-YFP iHcRed-t [4] generating pBMN-DD-HNF4α8. **2.** PCR of the myc-tag sequence was performed on the vector pcDNA5/FRT/TO/P2-HNF4α8 with the forward primer 5'-TGCCTTCAATTGCGCGCTATGGAGCAAAAG-3' and the reverse primer 5'-TGCCTTCAATTGGGTACCAGGCCTTGAATTC-3', introducing *MfeI* restriction sites on both sites of the tag. The *EcoRI* digested PCR fragment was introduced into the *EcoRI* site of pBMN-DD-HNF4α8 to generate pBMN-DD-myc-HNF4α8. **3.** For the final construct the DD-myc-HNF4α8 sequence was cloned as *BamHI-NotI* restriction fragment into the vector pcDNA5/FRT/TO/P2-HNF4α8 to replace myc-HNF4α8 and generate pcDNA5/FRT/TO/P2-DD-HNF4α8. The introduction of the mutation C106R into pcDNA5/FRT/TO/HNF4α2 was described elsewhere [1]. The corresponding mutation was introduced into the HNF4α8 variant of the pcDNA5/FRT/TO/P2-DD-HNF4α8 vector by replacing an *XhoI* restriction fragment spanning the mutational site with the appropriate *XhoI* sequence of pcDNA5/FRT/TO/HNF4α2-C106R.

The sequences of all final constructs as well as the presence of the mutation were verified by sequencing.

References of additional file 2:

1. Erdmann S, Senkel S, Arndt T, Lucas B, Lausen J, Klein-Hitpass L *et al.*: **Tissue-specific transcription factor HNF4alpha inhibits cell proliferation and induces apoptosis in the pancreatic INS-1 beta-cell line.** *Biol Chem* 2007, **388:** 91-106.

2. Thomas H, Jaschkowitz K, Bulman M, Frayling T, Mitchell SMS, Roosen S *et al.*: **A distant upstream promoter of the HNF-4α gene connects the transcription factors involved in maturity onset diabetes of the young.** *Hum Mol Genet* 2001, **10:** 2089-2097.

3. Banaszynski LA, Chen LC, Maynard-Smith LA, Ooi AG, Wandless TJ: **A rapid, reversible, and tunable method to regulate protein function in living cells using synthetic small molecules.** *Cell* 2006, **126:** 995-1004.

4. Chu BW, Banaszynski LA, Chen LC, Wandless TJ: **Recent progress with FKBP-derived destabilizing domains.** *Bioorg Med Chem Lett* 2008, **18:** 5941-5944.
